# Delineating the fecal microbiome of healthy domestic short-hair cats in South Korea

**DOI:** 10.3389/fvets.2025.1571107

**Published:** 2025-09-17

**Authors:** Hyun-Young Cho, Hyung-Joon Park, Jin-Sik Choi, Se-Hoon Kim, Min-Ok Ryu, Kyoung-Won Seo

**Affiliations:** ^1^Laboratory of Veterinary Internal Medicine, Department of Veterinary Clinical Science, College of Veterinary Medicine, Seoul National University, Seoul, Republic of Korea; ^2^UB Animal R&D Center, GRASSMEDI Co., Ltd., Seoul, Republic of Korea

**Keywords:** Korean short-hair cats, gut microbiome of healthy cats, fecal microbiome, 16S rRNA sequencing, body condition score, age-related differences, bacterial composition, diet

## Abstract

**Background:**

The gut microbiome is a vital component of an organism’s health, influencing metabolism, immune function, and overall homeostasis. In this study, we aimed to characterize the gut microbiota of healthy domestic short-hair cats in South Korea and evaluate the effects of age, body condition score (BCS), sex, and diet on microbial composition.

**Methods:**

From August to December 2023, 40 healthy cats aged 1–14 years with a body condition score (BCS) of 5–9 were selected. Cats were excluded if they had taken probiotics or antibiotics, exhibited gastrointestinal symptoms within the last 6 months, or had blood work abnormalities. DNA quantification was performed, and libraries targeting the V3 and V4 regions were prepared according to the Illumina 16S metagenomic sequencing protocol with a read length of 2 × 300 bp. The relative abundance of bacteria at the phylum, genus, and species levels was assessed according to the age, sex, diet, and BCS of the cats, with major bacterial groups selected for chart analysis.

**Results:**

Examination of the fecal samples from 40 healthy cats (aged 0.5–14 years) using 16S rRNA gene sequencing revealed 2,721 bacterial amplicon sequence variants. The predominant phyla were Bacillota, Bacteroidota, and Actinomycetota. Although age did not significantly affect alpha diversity, a trend toward increased diversity was observed in cats aged 7–14 years. *Phocaeicola* was more abundant in older cats, suggesting a possible association with age-related conditions. Furthermore, Verrucomicrobiota was enriched in cats with a BCS of 8–9, and *Ruminococcus torque* was positively correlated with higher BCS. Sex-based differences indicated increased levels of Pseudomonadota, *Finegoldia magna*, and *Sutterella massiliensis* in neutered males, potentially linked to inflammatory pathways. Dietary analysis revealed an increased abundance of *Blautia* and *Lachnoclostridium* following a combination of dry and wet food.

**Conclusion:**

Our findings provide critical insights into the core microbiota of domestic short-hair cats in South Korea, emphasizing the influence of geographic, physiological, and environmental factors on gut microbial diversity. The results offer a valuable foundation for advancing feline gut health research and enhancing health management strategies for felines, particularly in South Korea.

## 1 Introduction

The gut microbiota plays a pivotal role in maintaining an animal’s overall health and physiological functions by regulating key processes such as metabolism and immune modulation (1–3). During the metabolic activity of gut microbiota, short-chain fatty acids (SCFAs), including butyrate, propionate, and acetate, are produced through the fermentation of dietary fibers. These SCFAs influence host energy balance and lipid and glucose metabolism by interacting with various metabolic pathways in the liver and peripheral tissues (4). Gut microbial immune modulation is mediated by mechanisms wherein commensal microbes regulate systemic immune responses, mitigate inflammation, support long-term immune development through early-life microbiota interactions, prevent dysbiosis, and enhance anti-tumor immunity (5–7). Furthermore, the gut microbiome significantly impacts the digestive system and the health of various organs, including the immune system, skin, kidneys, brain, lungs, and liver, contributing to overall health and maintaining physiological balance across the body (8–13).

Considering these complex interactions, several factors including age, sex, diet, and environment shape the gut microbial landscape in dogs and cats, thereby influencing metabolic and immune functions (14–17). Several studies have investigated how factors like age, sex, body condition score (BCS), and diet relate to the diversity of gut microbiomes in domestic cats. One study demonstrated that overall alpha- and beta-diversity remain relatively stable across different age groups, though the number of core microbial taxa may decline with age (18). Regarding sex, no clear patterns have emerged indicating significant difference in microbial composition or diversity (19). Body condition appears to have a more pronounced influence; obese cats show decreased alpha diversity and distinct microbial composition compared to lean cats, indicating the impact of obesity on gut microbiome (20). Diet also play a critical role, as differences in macronutrient intake, such as protein-to-carbohydrate ratios, and feeding type have been linked to notable changes in microbial diversity and bacterial population (21, 22). The rising popularity of companion cats in South Korea has significantly increased their numbers, with domestic short-hair cats, commonly referred to as Korean short-hair (KSH) cats, being the most widely kept breed (23). Despite their prevalence, studies investigating the gut microbiome of healthy KSH cats are limited. Understanding the gut microbiome of this specific population is essential for developing targeted health management strategies and advancing knowledge in feline microbiology.

Characterizing the microbial composition of healthy cats in South Korea may facilitate a better understanding of microbiota shift relative to changes in the animal’s health. Consequently, in this study, we aimed to analyze the gut microbiota of healthy KSH cats living in South Korea and evaluate whether age, sex, BCS, and diet significantly impact its composition, providing insights into factors that influence the composition of the feline gut microbiome.

## 2 Materials and methods

### 2.1 Animals and metadata collection

All experimental procedures were approved by the Seoul National University Institutional Animal Care and Use Committee (approval number: SNU-231010-4) and adhered to the university’s ethical guidelines for the care and use of laboratory animals. This study was designed as a cross-sectional observational study to assess the gut microbiome of healthy KSH cats.

To identify suitable healthy cats, approximately 600 cat owners completed a detailed survey. The survey collected metadata on each cat’s identity, including name, age, neutering status, fecal consistency score, diet, gastrointestinal-related clinical signs, history of antibiotic or medication use, and the presence of comorbidities. Stool consistency was assessed using the 7-point Nestlé Purina Fecal Scoring System (1 = very hard/dry; 7 = watery diarrhea) (24). Scores of 2–3 were classified as normal (well-formed, pliable, segmented, and easy to pick up). For most cats, exact birth dates were unavailable; therefore, age was classified into categories (0.5–1 year, 2–6 years, and 7–14 years) based on owner-reported information.

Based on the survey responses, 50 cats were initially shortlisted. These cats subsequently underwent thorough physical examinations and hematological assessments. Following these evaluations, 40 cats that met the inclusion criteria were enrolled in the study. The inclusion criteria were as follows: (1) fecal score between 2 and 3; (2) body condition score between 4 and 9; (3) no gastrointestinal signs within the past 6 months; (4) no history of antibiotics, steroid, GI protectants, or probiotics administration within the past 6 months; (5) normal hematological and biochemical parameters; (6) up to date on vaccinations and parasite control; and (7) no evidence of systemic illness.

**Figure 1:**
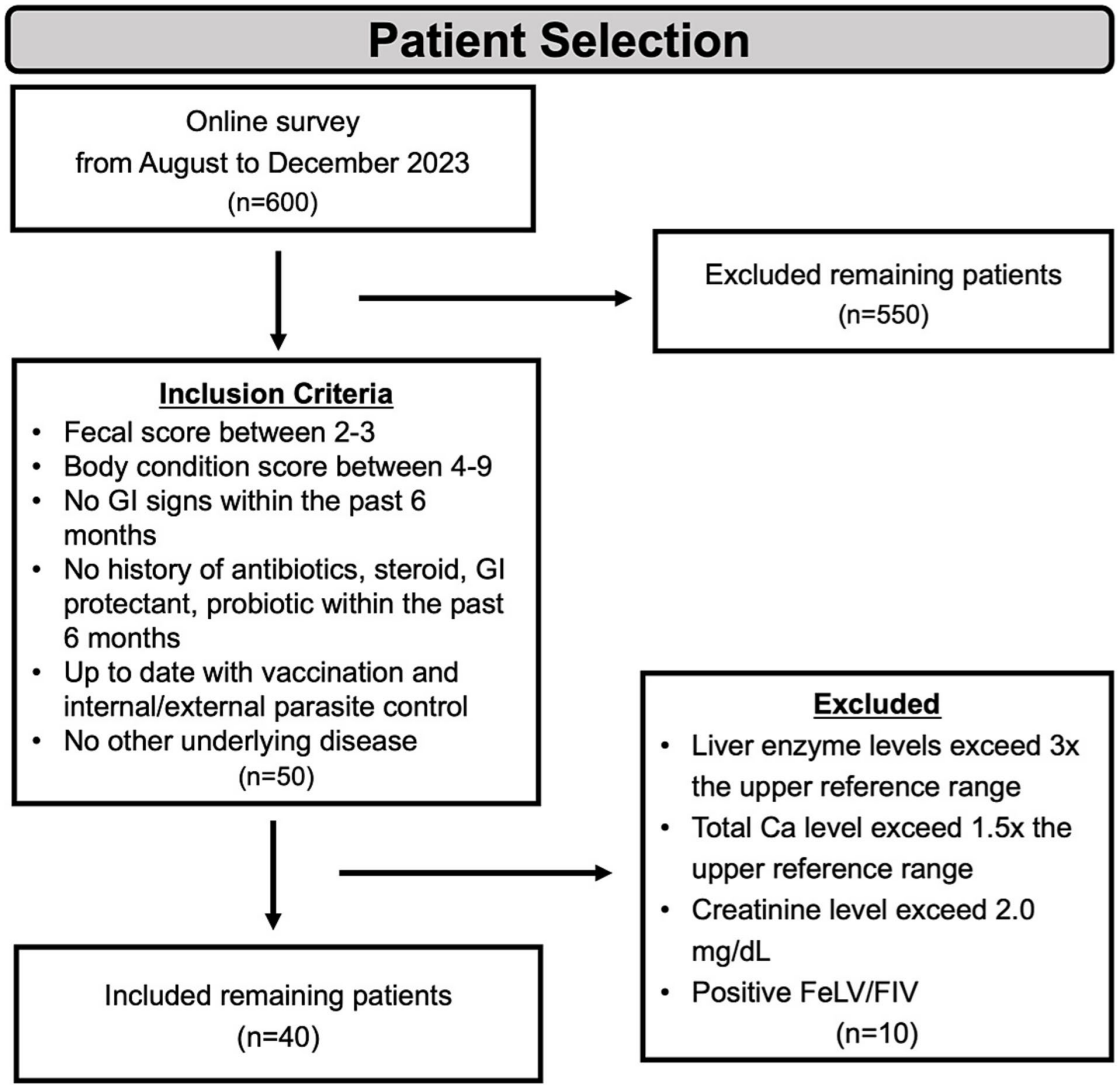
Scheme of animal selection. For the body condition score, a 9-point scale was used, while for the fecal core assessment, a 7-point scale was utilized. GI, gastrointestinal; BCS, body condition score; FeLV, feline leukemia virus; FIV, feline immunodeficiency virus.

The final 40 enrolled cats included 4 intact females, 13 spayed females, and 23 neutered males, aged 0.5–14 years, with a BCS range of 4 to 9. The BCS of each cat was assessed by a professional veterinarian (Figure 1).

### 2.2 Sample collection and DNA extraction

Participants in the study were provided with standardized kits to collect small fecal samples, which were subsequently returned by mail. Each kit included 2 mL screw-cap tubes containing 100% molecular-grade ethanol and silica beads to preserve the sample integrity during transportation. Upon arrival, the samples were transported to the laboratory and stored at temperatures below −80°C to ensure microbial DNA stability until DNA extraction.

DNA was extracted using the DNeasy PowerSoil Kit (Qiagen, Hilden, Germany), following the manufacturer’s protocol. Quantitative DNA analysis was performed using the Quant-IT PicoGreen assay (Invitrogen, Waltham, MA, United States).

### 2.3 Library construction and sequencing

To target the V3 and V4 hypervariable regions of the 16S rRNA gene, sequencing libraries were constructed following the Illumina 16S Metagenomic Sequencing Library Preparation protocol. During the first PCR, 5 ng of genomic DNA was amplified in a reaction mixture containing 5x reaction buffer, 1 mM dNTPs, 500 nM universal forward and reverse primers, and Herculase II fusion DNA polymerase. The thermal cycling conditions for the initial PCR involved denaturation at 95°C for 3 min and 25 amplification cycles at 95°C for 30 s, 55°C for 30 s, and 72°C at 30 s, followed by final extension at 72°C for 5 min. This initial amplification, using Illumina adapter overhangs, utilized a universal primer pair consisting of V3-F.: 5′-TCGTCGGCAGCGTCAGATGTGTATAAGAGACAGCCTACGGGNGGCWGCAG-3′ and V4-R: 5′-GTCTCGTGGGCTCGGAGATGTGTATAAGAGACAGGACTACHVGGGTATCTAATCC-3′. Following amplification, AMPure XP beads (Agencourt Bioscience, Beverly, MA) were used to purify the PCR products.

For the second PCR, the final sequencing library was constructed using Nextera XT Index primers with 2 μL of the purified product from the first PCR. The second PCR followed the same conditions as the above; instead, 25 cycles were used. PCR products were purified using AMPure XP beads to remove contaminants and ensure high-quality libraries.

Quantitative analysis of the purified library was conducted using qPCR following the KAPA Library Quantification Kit (KAPA Biosystems) protocol, optimized for Illumina platforms. The quality and size of the library were assessed using the TapeStation D1000 ScreenTape system (Agilent Technologies, Waldbronn, Germany). Paired-end sequencing (2 × 300 base pairs) was performed on the MiSeq™ platform (Illumina, San Diego, United States) by Macrogen (Seoul, South Korea), generating high-resolution data suitable for downstream microbial community analysis.

### 2.4 Gut microbial analysis

The raw sequencing data generated using the MiSeq platform were converted into FASTQ files using index sequences. Adapter and primer sequences were trimmed using Cutadapt (v3.2) with default parameter (25), whereas sequencing errors were corrected with DADA2 (v1.18.0) in R (v4.0.3) (26). Paired-end reads were merged, and chimeric sequences were identified and removed using the consensus method in DADA2 to generate high-resolution amplicon sequence variants (ASVs).

Taxonomic classification of ASVs was performed using BLAST+ (v2.9.0) (27) against the NCBI 16S Microbial Database, requiring a minimum of 85% query coverage and identity to ensure reliable classification. To standardize sequencing depth and reduce potential biases, subsampling was conducted using QIIME (v1.9) at a threshold of 47,916 reads per sample (28).

### 2.5 Statistical analysis

Microbial diversity (alpha diversity) within groups was calculated based on ASVs using the Shannon index, Inverse Simpson index (Gini-Simpson), Chao1 estimator, and Faith’s Phylogenetic Diversity (PD_whole_tree), all implemented in QIIME (v1.9). Unless otherwise specified, all values are reported as mean ± standard error of the mean (SEM). Beta diversity, reflecting differences in microbial community composition between groups, was assessed using weighted UniFrac distances. Principal coordinate analysis was used to visualize community composition. All statistical analyses were performed in R (v4.0.3) and visualized using ggplot2 (v3.2.1) for box plot generation. Predictor variables included age group (categorized as 0.5-1 year, 2–6 year, and 7–14 year), sex (neutered male and spayed female), BCS, and diet (dry food and dry + wet food). Statistical significance was set at *p* < 0.05, and all *p*-values were corrected for multiple comparisons using the Benjamini–Hochberg false discovery rate (FDR) method, unless otherwise specified.

Significant variations in microbial diversity indices and relative abundances were compared between groups using the Wilcoxon Rank Sum Test or Kruskal–Wallis test, followed by Dunn’s post-hoc test. Kruskal–Wallis tests were performed at both the phylum and genus levels to examine associations between bacterial taxon abundance and predictor variables, including age, sex, BCS, and diet. Beta diversity differences between groups were analyzed using UniFrac distance matrices and tested statistically by PERMANOVA.

The core microbiome was calculated by identifying taxa present in at least 50% of samples with a minimum relative abundance of 0.1%, to define prevalent and biologically meaningful microbial taxa representative of the healthy cat population studied.

## 3 Results

### 3.1 Core microbiome of healthy KSH cats

Fecal samples from 40 healthy KSH cats, aged 0.5–14 years and with a BCS 4–9, were analyzed for their gut microbiome composition (Table 1). A total of 2,721 bacterial ASVs, classified into 15 phyla, 25 classes, 47 orders, 96 families, 299 genera, and 556 species were identified.

**Table 1:** Twenty-two core microbiome genera identified in healthy KSH cats.

Phylum	Class	Order	Family	Genus	Mean	SEM	Median	IQR
Bacteroidota	Bacteroidia	Bacteroidales	Prevotellaceae	Segatella	9.35	2.03	1.34	18.17
Bacillota	Clostridia	Eubacteriales	Lachnospiraceae	Blautia	9.15	1.37	7.22	9
Bacteroidota	Bacteroidia	Bacteroidales	Bacteroidaceae	Phocaeicola	8.43	1.52	5.2	9.77
Bacteroidota	Bacteroidia	Bacteroidales	Bacteroidaceae	Bacteroides	6.06	1.28	2.91	6.21
Bacillota	Clostridia	Eubacteriales	Peptostreptococcaceae	Peptacetobacter	5.14	1.17	2.54	5.46
Actinomycetota	Coriobacteriia	Coriobacteriales	Coriobacteriaceae	Collinsella	4.39	0.85	2.47	4.79
Bacillota	Tissierellia	Tissierellales	Peptoniphilaceae	Anaerococcus	4.34	1.39	0	4.96
Bacillota	Negativicutes	Selenomonadales	Selenomonadaceae	Megamonas	3.73	0.91	0.48	6.03
Pseudomonadota	Gammaproteobacteria	Enterobacterales	Enterobacteriaceae	Escherichia	3.47	1.06	0.07	3.99
Fusobacteriota	Fusobacteriia	Fusobacteriales	Fusobacteriaceae	Fusobacterium	3.42	0.68	1.32	4.87
Actinomycetota	Actinomycetes	Bifidobacteriales	Bifidobacteriaceae	Bifidobacterium	2.97	1.29	0.23	1.08
Bacteroidota	Bacteroidia	Bacteroidales	Porphyromonadaceae	Porphyromonas	2.94	1.23	0	0.01
Bacillota	Tissierellia	Tissierellales	Peptoniphilaceae	Peptoniphilus	2.79	0.77	0	4.08
Bacillota	Erysipelotrichia	Erysipelotrichales	Coprobacillaceae	Catenibacterium	2.37	0.77	0	2.78
Bacillota	Clostridia	Eubacteriales	Lachnospiraceae	Mediterraneibacter	2.19	0.41	1.43	1.65
Bacillota	Tissierellia	Tissierellales	Peptoniphilaceae	Finegoldia	2.17	0.74	0	0.28
Pseudomonadota	Betaproteobacteria	Burkholderiales	Sutterellaceae	Sutterella	2.02	0.5	0.74	2.47
Bacillota	Clostridia	Eubacteriales	Clostridiaceae	Clostridium	1.34	0.35	0.47	1.55
Bacillota	Clostridia	Eubacteriales	Lachnospiraceae	Lachnoclostridium	1.28	0.14	1.31	1.35
Bacillota	Erysipelotrichia	Erysipelotrichales	Erysipelotrichaceae	Holdemanella	1.27	0.61	0	0.89
Bacillota	Negativicutes	Veillonellales	Veillonellaceae	Megasphaera	1.12	0.28	0.05	1.68
Campylobacterota	Epsilonproteobacteria	Campylobacterales	Helicobacteraceae	Helicobacter	1.08	0.47	0	0.41
Core microbiome total					81.03	1.55	82.74	10.19

Values are expressed as % relative abundance (mean ± SEM; median and IQR).

**Figure 2:**
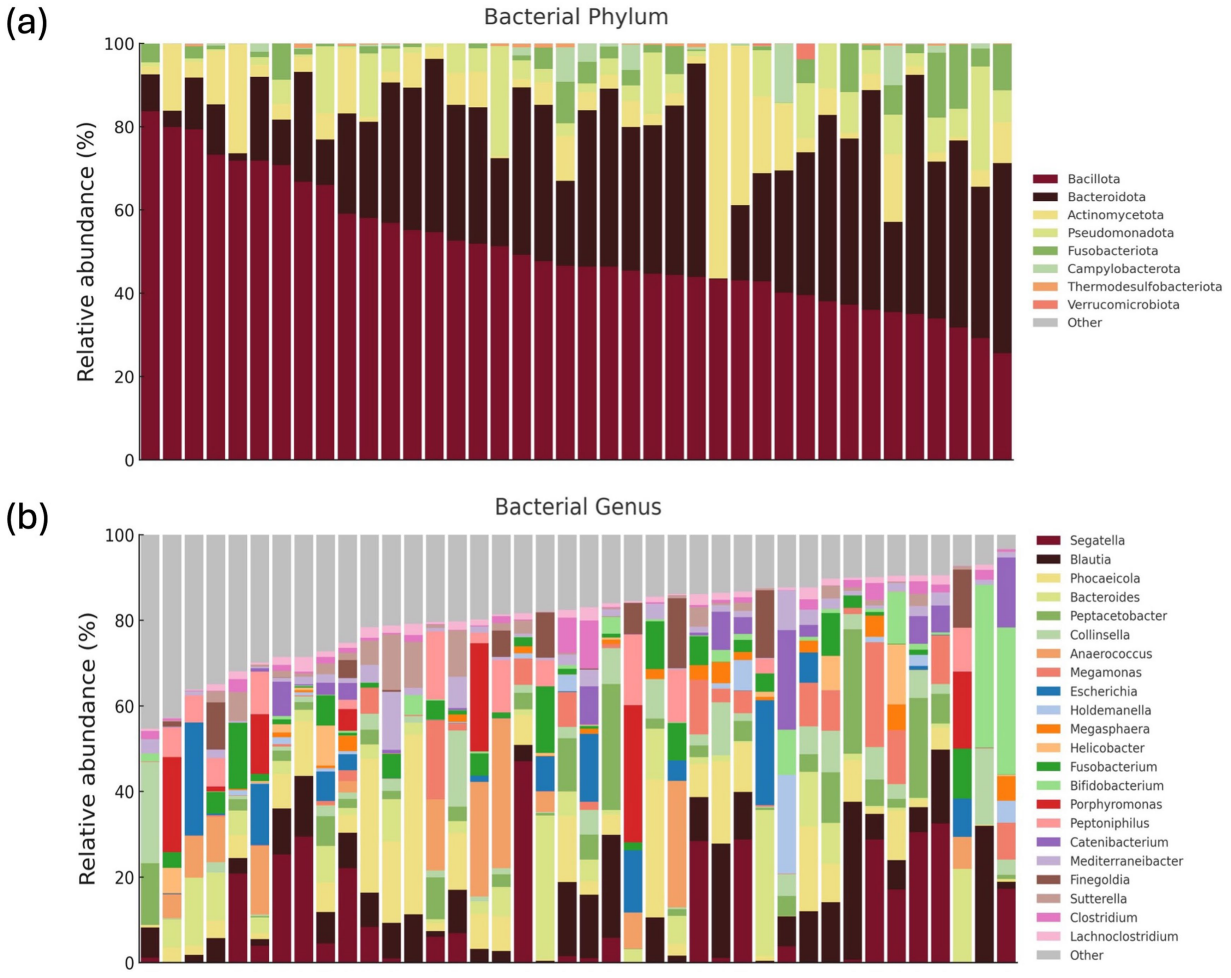
Gut microbiome composition of 40 healthy KSH cats. Comprehensive gut microbiome composition of 40 healthy KSH cats. Relative abundances of phyla accounting for 0.01% or more of the total sequences **(a)**. Relative abundances of genera accounting for 1% or more of the total sequences from the eight major phyla **(b)**.

The predominant bacterial phyla (≥1% of total sequences) were Bacillota (50%), Bacteroidota (30%), Actinomycetota (8%) at the phylum level (Figure 2 and Supplementary Table S1), with other major phyla including Pseudomonadota, Fusobacteriota, and Campylobacterota. The overall alpha diversity metrics for the 40 healthy KSH cats showed a mean Chao1 index of 245.7 (±89.3) and a mean Shannon diversity index of 4.8 (±0.7), indicating substantial microbial richness and diversity within this cohort (Supplementary Table S1).

At the genus level, 22 taxa were identified as predominant (≥1% of total sequences) bacterial genera (Figure 2), with the top five most abundant genera being Segatella, Blautia, Phocaeicola, Bacteroides, and Peptacetobacter. At the species level, 28 predominant species were identified, including *Segatella copri* (formerly *Prevotella corp*i), *Peptacetobacter hiranonis, Megamonas funiformis*, *Escherichia fergusonii, Collinsella intestinalis*, and *Anaerococcus octavius* (Supplementary Table S1).

**Figure 3:**
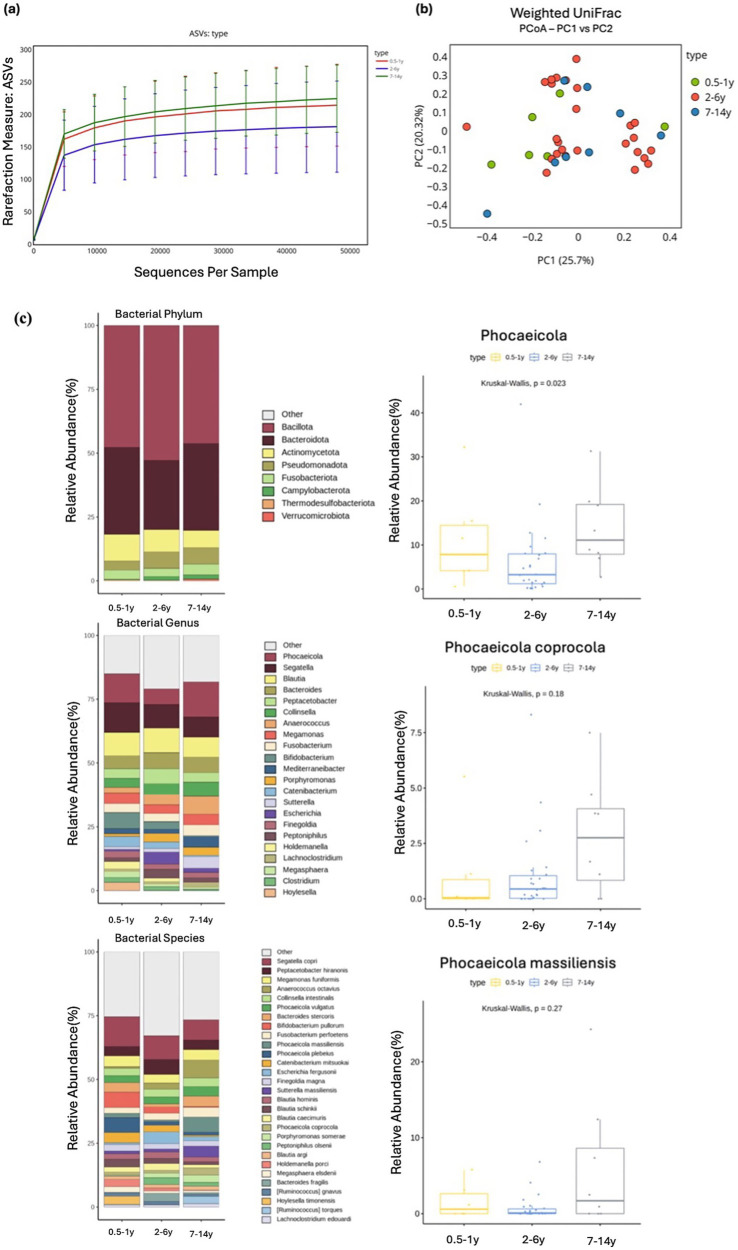
Alpha and beta diversity were analyzed based on different age groups, with microbial richness assessed through ASV. **(a)** PCoA was conducted using weighted UniFrac distance. **(b)** The relative abundances of bacteria at the phylum, genus, and species levels were analyzed based on the age of the cats. Major bacterial groups were represented through chart analysis (phylum ≥ 0.01%, genus ≥ 1.0%, species ≥ 1.0% of total abundance). **(c)** Box plots were used to illustrate bacterial groups that showed significant differences, and box plots with *p* > 0.05 were shown to indicate the tendency of increase with age.

**Figure 4:**
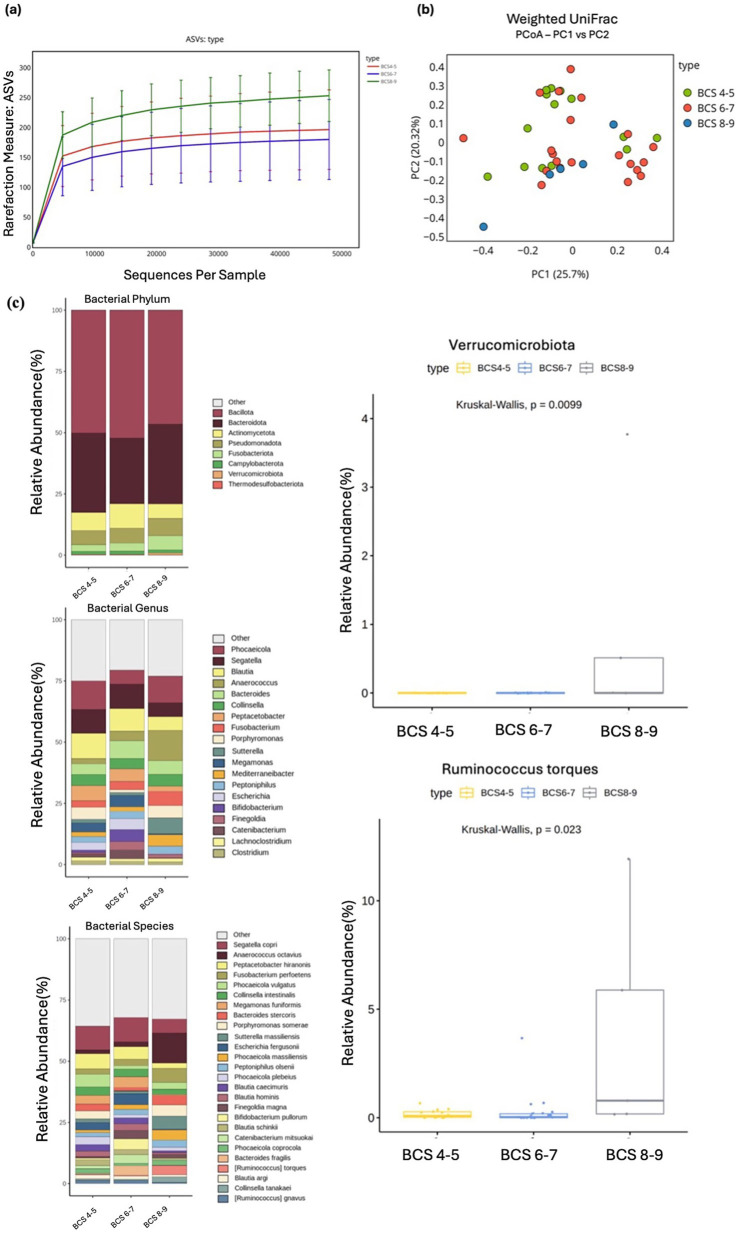
Alpha and beta diversity were analyzed based on different BCS groups, with microbial richness assessed through ASV. **(a)** PCoA was conducted using weighted UniFrac distance. **(b)** The relative abundances of bacteria at the phylum, genus, and species levels were analyzed based on the BCS of the cats. Major bacterial groups were represented through chart analysis (phylum ≥ 0.01%, genus ≥ 1.0%, species ≥ 1.0% of total abundance). **(c)** Box plots were used to illustrate bacterial groups that showed significant differences.

**Figure 5:**
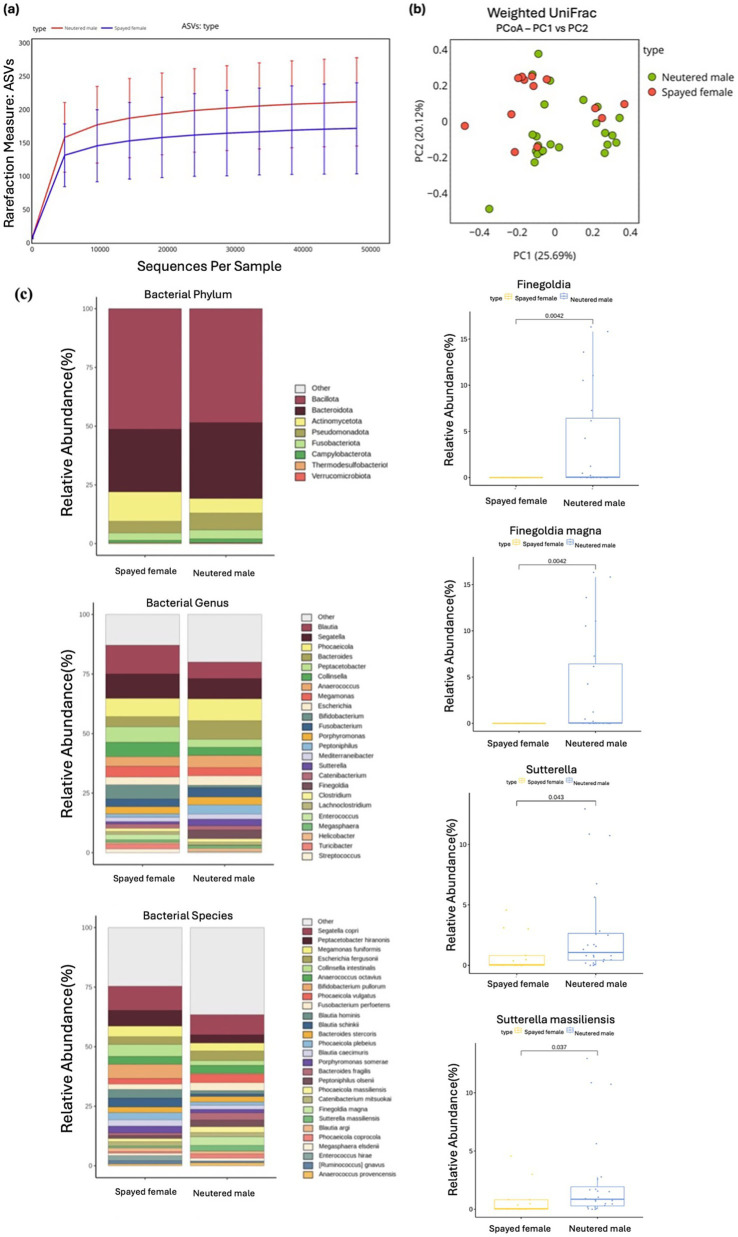
Alpha and beta diversity were assessed according to different sex groups, with microbial richness evaluated using ASV. **(a)** Weighted UniFrac distance was used to conduct the PCoA analysis. **(b)** The relative abundances of bacteria were evaluated at the phylum, genus, and species levels based on the sex of the cats. Major bacterial groups were visualized through charts, including phyla (≥0.01%), genera (≥1.0%), and species (≥1.0%) representing total abundance. **(c)** Box plots were utilized to depict bacterial groups that exhibited significant differences.

**Figure 6:**
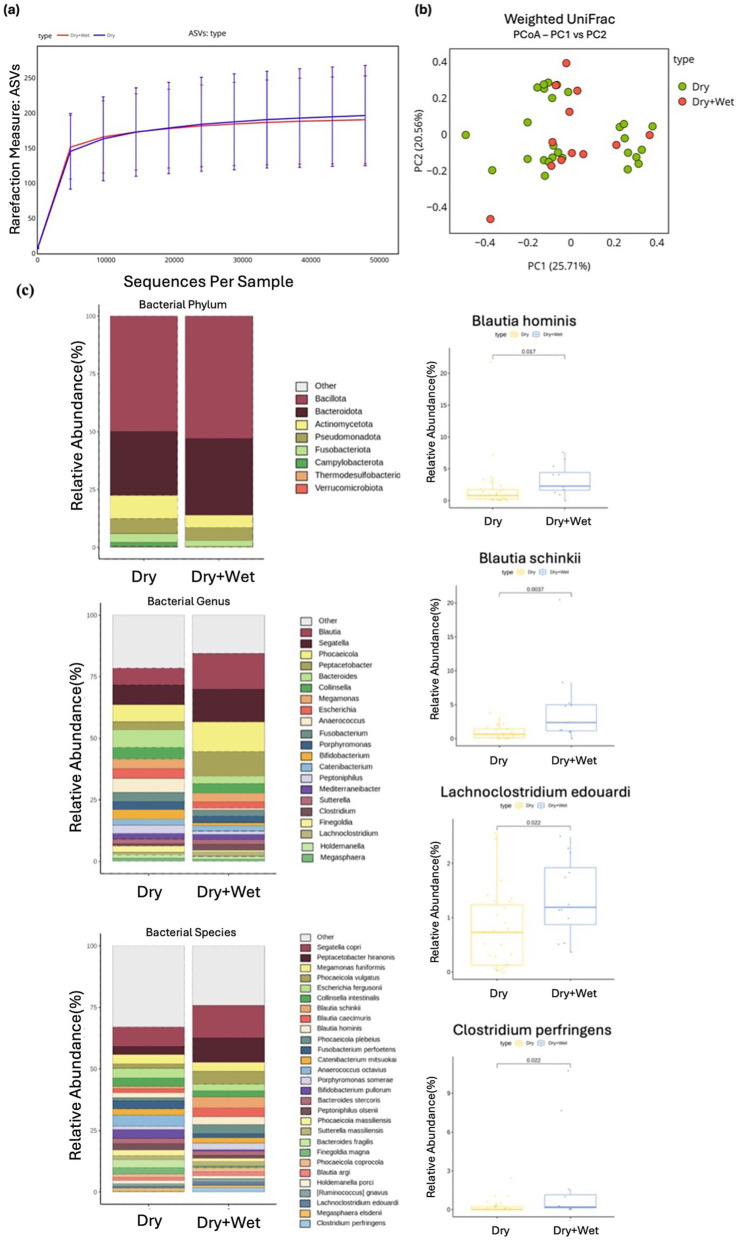
Alpha and beta diversity were examined across different diet groups, with microbial richness determined through ASV. **(a)** PCoA was performed using weighted UniFrac distance. **(b)** The relative abundances of bacteria were evaluated at the phylum, genus, and species levels based on the diet of the cats. Major bacterial groups were depicted using chart analysis, including phyla (≥0.01%), genera (≥1.0%), and species (≥1.0%) of the total abundance. **(c)** Box plots were utilized to present bacterial groups that showed significant differences.

The general characteristics of the gut microbiome in all 40 healthy cats are presented (Figure 2), which shows the relative abundances of major phyla and genera. In addition, the gut microbial composition stratified by age, BCS, sex, and diet is illustrated (Figures 3–6; Supplementary Figures S1–S4). Detailed abundance data are provided (Supplementary Tables S1–S5).

### 3.2 Age

Comparative analysis across age groups, categorized as 0.5–1 year, 2–6 year, and 7–14 years, revealed no significant differences in alpha diversity measures (ASVs, Shannon, Gini-Simpson, PD_whole_tree). However, most indices exhibited a trend toward increased microbial diversity with age (Figure 3A). Similarly, beta diversity analysis indicated no significant changes in the microbial community structure, and no clear clustering patterns were observed according to age (Figure 3B). Although no significant differences were detected at the phylum level, Verrucomicrobiota exhibited a trend of increasing abundance with age (Kruskal–Wallis test, *p* = 0.095). At the genus level, a significant difference was observed for *Phocaeicola* (Kruskal–Wallis test, *p* = 0.023), with cats aged 7–14 years harboring its higher abundance compared with those aged 2–6 years. By contrast, *Segatella* showed a non-significant decrease in abundance with age (Kruskal–Wallis test, *p* = 0.695; Supplementary Table S2). At the species level, none of the taxa exhibited significant differences after correction for multiple testing. However, some notable trends were observed. *Phocaeicola vulgatus* (Kruskal–Wallis test, *p* = 0.357) and *Phocaeicola coprocola* (Kruskal–Wallis test, *p* = 0.344) tended to increase with age, whereas *Bacteroides stercoris* demonstrated a trend toward decreased abundance (Kruskal–Wallis test, *p* = 0.057; Figure 3C).

### 3.3 BCS

Neither alpha nor beta diversity measures demonstrated significant differences between the BCS groups. However, an increasing trend in diversity was observed for the ASV index (Kruskal–Wallis test, *p* = 0.054; Figures 4A, B). At the phylum level, Verrucomicrobiota exhibited a significant increase in abundance in the BCS 8–9 group compared with other groups (Kruskal–Wallis test, *p* = 0.009). At the genus level, *Phocaeicola* displayed a higher abundance in the BCS 8–9 group (Kruskal–Wallis test, *p* = 0.057), whereas *Segatella* exhibited a decrease with higher BCS (Kruskal–Wallis test, *p* = 0.754), although neither finding was significant. At the species level, several taxa showed patterns associated with BCS. Notably, *Ruminococcus gnavus* was significantly more abundant in the BCS 8–9 group compared with those with lower BCS (Kruskal–Wallis test, *p* = 0.023), suggesting a positive correlation with body condition (Figure 4C).

Other species exhibited trends, though not significant, with *Bacteroides stercoris* decreasing in abundance as BCS increased (Kruskal–Wallis test, *p* = 0.117), whereas *Phocaeicola copri* (Kruskal–Wallis test, *p* = 0.344) and *Phocaeicola vulgatus* (Kruskal–Wallis test, *p* = 0.357) demonstrated slight increases in abundance with higher BCS (Supplementary Table S3).

### 3.4 Sex

Alpha and beta diversity measures showed no significant differences between spayed females and neutered males, indicating similar microbial richness and evenness between the two groups (Figures 5A, B). At the phylum level, Pseudomonadota demonstrated a higher relative abundance in neutered males compared with spayed females, with marginal statistical significance (Wilcoxon rank-sum test, *p* = 0.050).

At the genus level, *Sutterella* (Wilcoxon rank-sum test, *p* = 0.043) and *Finegoldia* (Wilcoxon rank-sum test, *p* = 0.004) were significantly more abundant in the neutered male group than in the spayed female group. At the species level, several taxa exhibited differences between the groups. Specifically, *Finegoldia magna* (Wilcoxon rank-sum test, p = 0.004) and *Sutterella massiliensis* (Wilcoxon rank-sum test, *p* = 0.037) were significantly more abundant in neutered males compared with spayed females (Figure 5C).

### 3.5 Diet

No significant differences were observed in alpha or beta diversity measures between the dry diet group and the dry + wet diet group. Similarly, no statistical differences were observed in the phylum-level composition of the intestinal microbial community between the two diet groups (Figures 6A, B).

At the genus level, two taxa exhibited significant differences in abundance between the groups. The dry + wet diet group showed a significantly higher abundance of *Blautia* (Wilcoxon rank-sum test, *p* = 0.003) and *Lachnoclostridium* (Wilcoxon rank-sum test, *p* = 0.039) compared with that detected in the dry diet group (Supplementary Table S5). At the species level, several taxa demonstrated significant differences between the two diet groups. *Blautia hominis* (Wilcoxon rank-sum test, *p* = 0.017), *Blautia schinkii* (Wilcoxon rank-sum test, *p* = 0.003), *Lachnoclostridium edouardi* (Wilcoxon rank-sum test, *p* = 0.022), and *Clostridium perfringens* (Wilcoxon rank-sum test, p = 0.022) were significantly more abundant in the dry + wet diet group compared with the dry diet group (Figure 6C).

## 4 Discussion

Studies conducted in various countries have demonstrated that geographic location significantly influences gut microbiome composition, resulting in differences at both the phylum and genus levels. Previous research in healthy domestic cats from the United States identified Bacillota, Bacteroidota, and Actinomycetota as the predominant phyla, with *Prevotella*, *Bacteroides*, *Collinsella*, *Blautia*, and *Megasphaera* being the dominant genera (18, 29). Similarly, studies conducted in the United Kingdom reported that the core microbiome largely comprises the phyla Bacillota and Bacteroidetes, with *Bacteroides* and *Prevotella* as the predominant genera (30).

In the present study, Bacillota made up 50% of the total microbiome, whereas Bacteroidota was 30% and Actinomycetota was 8% at the phylum level, which is similar to previous findings (18, 29). However, differences in genus-level composition were observed. While earlier studies emphasized genera such as *Segatella*, *Bacteroides*, *Collinsella, Blautia*, and *Megasphaera*, this study identified *Segatella* (9.3%), *Blautia* (9.1%), *Phocaeicola* (8.4%), *Bacteroides* (6.0%), and *Peptacetobacter* (5.1%) as the dominant genera. These findings underscore both the consistency in core microbiota composition across studies and the regional variation driven by environmental factors such as geography, diet, and husbandry practices.

Biological aging, estimated from frailty or physiological markers, has been linked to lower microbial diversity and richness, greater abundances of frailty-associated bacteria, impaired SCFA pathways, and reduced gut stability, irrespective of chronological age (31). In the present study, we assessed age in years only when analyzing feline gut microbiome changes. Notably, our findings are consistent with previous research showing no significant association between age and alpha-or beta-diversity in cats (18).

Studies on age-related gut microbiome changes in cats have reported mixed findings influenced by health status, diet, and external factors (32, 33). For instance, a previous Japanese study analyzing five different age groups (juvenile, weaning, adolescent, adult, and elderly) observed a decrease in microbial diversity during adolescence and adulthood, followed by an increase in elderly cats, suggesting a unique reorganization of microbial communities during feline aging (34). This trend contrasts with the steady decline observed in human studies, highlighting the different physiological aging patterns in cats.

In our study, although no significant differences in alpha diversity were observed, a decline in diversity was noted from 0–1 years to 2–6 years, followed by a slight increase in the 7–14 year age group. This pattern aligns with prior findings, suggesting that the initial decline could be attributed to the stabilization of the gut microbiome during adulthood, whereas the subsequent increase may reflect microbial adaptation to aging (34). Therefore, the feline gut microbiome undergoes unique adaptive reorganizations with age. Nonetheless, further comparative analyses among specific feline age groups are necessary to better understand microbiome composition shifts.

Previous research on obese cats in the United States reported a significant decrease in microbial diversity, with a reduction in the Firmicutes/Bacteroidetes ratio and increased relative abundances of *Bifidobacterium* and *Dialister* at the genus level and of *Olsenella provencensis* and *Campylobacter upsaliensis* at the species level (15).

In contrast, our study did not observe a significant increase in the Firmicutes/Bacteroidetes ratio relative to BCS. Instead, the abundance of specific microbiota such as *Ruminococcus torques* increased with higher BCS, which has been implicated in the degradation of mucin and enhancement of energy harvest and fat accumulation (35). These findings indicate that *Ruminococcus torques* may serve as an indicator of obesity-related gut microbiota changes in cats, highlighting the potential for targeted microbial interventions in managing obesity, though further research is needed to support these observations. Additionally, geographic factors may influence the composition and behavior of obesity-associated gut microbiome.

A study conducted in the United Kingdom reported no significant sex-specific differences in gut microbiome composition between intact male and female cats when environmental factors were controlled, which contrasts with the findings of our study (19).

In the present study, the relative abundances of *Sutterella massiliensis* and *Finegoldia magna* were increased in neutered male cats. Although research on *Sutterella massiliensis* is limited, members of the genus *Sutterella* may exhibit pro-inflammatory properties. Similarly, *Finegoldia magna* is recognized in human medicine as an opportunistic pathogen capable of inducing inflammation (36, 37). Experimental studies in mice have also reported proliferation of inflammation-inducing gut microbiota following reductions in testosterone levels after castration (38). Although testosterone levels were not measured in our study, the observed increase in inflammation-associated microbiota in neutered males suggests that factors beyond testosterone may contribute to inflammatory microbiome shifts in neutered cats. These findings warrant further investigation into the role of sex hormones and other contributing factors in shaping the gut microbiome in neutered cats.

Diet significantly influences gut microbiota composition in humans, with high-fiber diets increasing butyrate-producing bacteria, which contribute to SCFA production and improved gut health in humans (39). In our study, the relative abundance of *Blautia* was significantly higher in the dry + wet group. *Blautia*, as a butyrate-producing bacterium, exhibits an anti-inflammatory effect that is crucial in gut health, and its higher abundance may indicate increased SCFA production (40). At the species level, *Blautia hominis* and *Blautia schinkii* were significantly high in the dry + wet group. Similarly, *Clostridium perfringens*, a potential pathogen capable of causing gastrointestinal symptoms, was also more prevalent in cats on a wet + dry diet. However, as *Clostridium perfringens* is commonly detected in healthy cats without diarrhea, it may still be considered part of the normal gut microbiota (41).

Previous studies conducted across different states in the United States have reported that commercial dry and wet food diets differ in their protein, carbohydrate, and fat ratios, which can promote the growth of specific bacteria and alter the gut microbiome composition (22, 32, 42). Similarly, in our study, dietary differences also resulted in variations in the relative abundance of the gut microbiome. Our findings suggest that dry + wet food diets may support a healthier gut microbiome by promoting beneficial butyrate-producing bacteria. However, further research is warranted to explore the long-term health impacts of these microbiome changes and the role of dietary variations. The higher abundance of beneficial bacteria with combined wet and dry food suggests that moisture levels may influence microbial composition. Additionally, geographic factors may influence the composition and behavior of obesity-associated gut microbiome. Previous studies have consistently demonstrated differences in the gut microbiota composition of healthy companion animals based on whether they are fed kibble or non-kibble diets (18). In the present study, all enrolled cats were fed commercial kibble as part of their diet, which may have limited our ability to detect microbiome differences associated with dietary type. Notably, the higher abundance of beneficial bacteria in cats fed a combination of wet and dry food suggests that dietary moisture levels may also influence microbial composition. This highlights the need to investigate the effects of varying dietary moisture content. Furthermore, future studies are warranted to explore the long-term health implications of these microbiome changes and the role of dietary variation.

### 4.1 Limitations

The limitations of the present study are as follows: First, the study involved 40 cats, which is a relatively small sample size, and thus, the results may not be highly generalizable. However, in previous studies showing differences between diseased and healthy groups, the number of healthy cats was smaller than in our study. Therefore, the healthy cats in this study could serve as a reference group for future comparisons with diseased groups. Second, in most cases, the exact age of the cats could not be confirmed. Age information obtained from owner reports was therefore classified into three broad categories (0.5–1 year, 2–6 years, and 7–14 years). This approach prevented the use of age as a continuous variable and limited further subdivision of the existing ranges, which may have reduced the ability to detect subtle age-related variations in the microbiome. Sample sizes were unequal across age groups. In addition, covariates co-varied with age: all cats in the 7–14-year group were fed a dry-only diet, had higher BCS, and were spayed or neutered; this constrains our ability to isolate age effects from diet, BCS, and sex status. Third, health status was determined by questionnaire and screening, and detailed dietary monitoring was not performed. These factors may have contributed to the variability observed in the microbiome. In addition, the cross-sectional design provides only a snapshot of the gut microbiota at a single time point, preventing causal inference or assessment of temporal dynamics. Fourth, the voluntary, survey-based recruitment process influenced the final sample size and demographic composition. Most enrolled cats were neutered males, reflecting regional ownership trends rather than intentional selection. Although this allowed comparison between neutered males and females, intact animals were under-represented. Future studies should specifically evaluate the combined effects of sex and neuter status in larger, more balanced cohorts. Fifth, dietary information was obtained from owners, and comprehensive nutritional profiles (e.g., macronutrient and fiber content) were unavailable for all commercial products. The main diet types are summarized in Table 2, but the lack of granular composition data limits interpretation. Future investigations should gather detailed nutrient information and explore how different diet formulations—particularly fiber-rich diets—affect microbial diversity across age groups and breeds. Finally, the small sample size and absence of longitudinal data make it difficult to draw firm conclusions about relationships among diet, age, body condition, and microbial composition. Long-term, longitudinal studies with larger and more diverse populations are needed to track microbiome shifts over time and clarify causal links between dietary factors, microbial changes, and feline health outcomes.

**Table 2:** Characteristics of the 40 healthy cats in the reference set.

Age group	*n*	Age (months)	Body condition score	Sex			Diet	
				F (%)	SF (%)	MN (%)	Dry (%)	Dry + wet (%)
0.5–1 y	6	10.2 (±3.03)	5.67 (±1.22)	17	50	33	40	60
2–6 y	26	35.5 (±16.11)	5.92 (±0.93)	8	27	65	65	35
7–14 y	8	129 (±21.99)	7.13 (±1.25)	0	37	63	100	0

BCS, body condition score; F (%), percentage of female cats in the age group; SF (%), percentage of spayed female cats in the age group; MN (%), percentage of neutered male cats in the age group; dry (%), percentage of cats fed only dry food; dry + wet (%), percentage of cats fed both dry and wet food.

## 5 Conclusion

We aimed at identifying the core microbiome of KSH cats—the most common breed living in South Korea—and analyzed the variations according to sex, age, BCS, and diet. While the core microbiome was defined by Bacillota, Bacteroidota, and Actinomycetota, distinct differences were observed at the genus level, including the distribution of *Segatella*, *Blautia*, *Phocaeicola*, *Bacteroides*, and *Peptacetobacter*, potentially representing a reference dataset of microbiome samples taken from healthy cats living in South Korea. Additionally, differences at the genus level in the core microbiome were observed compared with those in other countries, along with variations in the gut microbiome with respect to age, BCS, sex, and diet factors. Furthermore, this study suggests that future research on the gut microbiome and the gut–organ axis should also consider geographical background. Future research should further explore the role of environmental factors in shaping the feline gut microbiome, particularly focusing on the impact of diet and region-specific variables, to develop more targeted health interventions for cats. Overall, this study provides crucial insights into the microbiome of KSH cats, contributing to a deeper understanding of feline gut health and the factors that influence it.

## Data Availability

The sequencing data generated for this study are available in the NCBI Sequence Read Archive (SRA) under BioProject accession number PRJNA1322165.
