# Supplementary material for: Delineating the fecal microbiome of healthy domestic short-hair cats in South Korea
Source: Front Vet Sci. 2025 Sep 17;12:1571107. doi: 10.3389/fvets.2025.1571107 (PMC12486308; doi:10.3389/fvets.2025.1571107)
Supplement: Supplementary file 1 [file Supplementary_file_1.docx]

Supplementary Material

## Supplementary Tables

Supplementary Table S1. Relative abundance of gut microbiome in 40 healhty cats. Major bacterial group (Phylum ≥0.1%, Genus ≥1%, Species ≥1%) are shown.

|  | **Mean** | **SEM** |
| --- | --- | --- |
| **Phylum** | | |
| *Bacillota* | 50.7 | 2.4 |
| *Bacteroidota* | 29.5 | 2.3 |
| *Actinomycetota* | 8.6 | 1.7 |
| *Pseudomonadota* | 6.0 | 1.0 |
| *Fusobacteriota* | 3.4 | 0.7 |
| *Campylobacterota* | 1.3 | 0.5 |
| *Thermodesulfobacteriota* | 0.2 | 0.1 |
| *Verrucomicrobiota* | 0.1 | 0.1 |
| **Genus** | | |
| *Segatella* | 9.3 | 2.0 |
| *Blautia* | 9.2 | 1.4 |
| *Phocaeicola* | 8.4 | 1.5 |
| *Bacteroides* | 6.1 | 1.3 |
| *Peptacetobacter* | 5.1 | 1.2 |
| *Collinsella* | 4.4 | 0.9 |
| *Anaerococcus* | 4.3 | 1.4 |
| *Megamonas* | 3.7 | 0.9 |
| *Escherichia* | 3.5 | 1.1 |
| *Fusobacterium* | 3.4 | 0.7 |
| *Bifidobacterium* | 3.0 | 1.3 |
| *Porphyromonas* | 2.9 | 1.2 |
| *Peptoniphilus* | 2.8 | 0.8 |
| *Catenibacterium* | 2.4 | 0.8 |
| *Mediterraneibacter* | 2.2 | 0.4 |
| *Finegoldia* | 2.2 | 0.7 |
| *Sutterella* | 2.0 | 0.5 |
| *Clostridium* | 1.3 | 0.4 |
| *Lachnoclostridium* | 1.3 | 0.1 |
| *Holdemanella* | 1.3 | 0.6 |
| *Megasphaera* | 1.1 | 0.3 |
| *Helicobacter* | 1.1 | 0.5 |
| **Species** | | |
| *Segatella copri* | 9.3 | 2.0 |
| *Peptacetobacter hiranonis* | 5.1 | 1.2 |
| *Megamonas funiformis* | 3.6 | 0.9 |
| *Escherichia fergusonii* | 3.4 | 1.1 |
| *Collinsella intestinalis* | 3.1 | 0.7 |
| *Anaerococcus octavius* | 3.1 | 1.3 |
| *Phocaeicola vulgatus* | 2.9 | 1.1 |
| *Fusobacterium perfoetens* | 2.8 | 0.6 |
| *Bifidobacterium pullorum* | 2.6 | 1.3 |
| *Catenibacterium mitsuokai* | 2.4 | 0.8 |
| *Blautia hominis* | 2.3 | 0.6 |
| *Blautia caecimuris* | 2.3 | 0.5 |
| *Bacteroides fragilis* | 2.2 | 1.1 |
| *Finegoldia magna* | 2.2 | 0.7 |
| *Bacteroides stercoris* | 2.2 | 0.5 |
| *Peptoniphilus olsenii* | 2.1 | 0.6 |
| *Blautia schinkii* | 2.0 | 0.5 |
| *Phocaeicola plebeius* | 2.0 | 0.6 |
| *Phocaeicola massiliensis* | 1.9 | 0.7 |
| *Porphyromonas somerae* | 1.8 | 1.0 |
| *Sutterella massiliensis* | 1.8 | 0.5 |
| *Phocaeicola coprocola* | 1.4 | 0.3 |
| *Blautia argi* | 1.4 | 0.2 |
| *Holdemanella porci* | 1.2 | 0.6 |
| *Ruminococcus gnavus* | 1.2 | 0.2 |
| *Megasphaera elsdenii* | 1.1 | 0.3 |
| *Helicobacter bilis* | 1.0 | 0.5 |
| *Anaerococcus provencensis* | 1.0 | 0.5 |

Supplementary Figure S1: Alpha and beta diversity according to age. (a) Microbial richness and evenness were measured based on Shannon index. PCoA was performed based on unweighted and weighted UniFrac distances. (b) List of alpha diversity indices. (c)

1. **(b)**


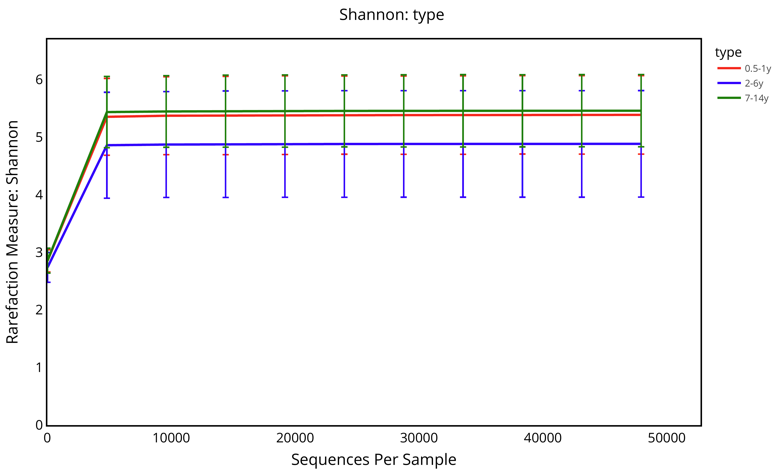

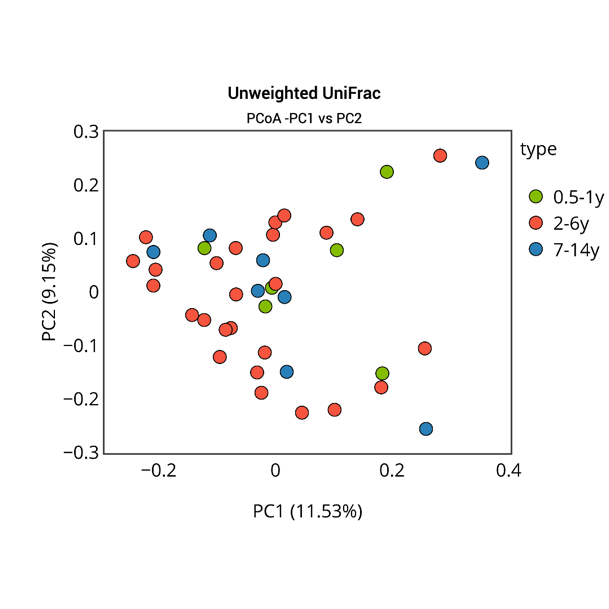


**(c)**

| **Diversity Index** | **0.5-1y** | | **2-6y** | | **7-14y** | | **P-value** | **FDR** |
| --- | --- | --- | --- | --- | --- | --- | --- | --- |
|  | **Mean** | **SEM** | **Mean** | **SEM** | **MEAN** | **SEM** |  |  |
| ASVs | 214.67 | 25.74 | 181.58 | 13.78 | 224.75 | 18.33 | 0.17 | 0.37 |
| Shannon | 5.39 | 0.28 | 4.88 | 0.18 | 5.46 | 0.22 | 0.19 | 0.37 |
| Gini-Simpson | 0.94 | 0.01 | 0.92 | 0.01 | 0.94 | 0.01 | 0.30 | 0.37 |
| PD whole tree | 20.39 | 1.88 | 18.17 | 1.06 | 20.37 | 1.11 | 0.37 | 0.37 |

Supplementary Table S2. Relative abundance at the phylum, genus, and species level according to age. Kruskal–Wallis test was used, and data are shown as the mean ± SEM. Major or significantly different bacterial groups (abundance ≥ 1% or P < 0.05) are shown. Asterisks indicate P < 0.05.

|  | **0.5-1y** | **2-6y** | **7-14y** |  | |
| --- | --- | --- | --- | --- | --- |
|  | **Mean**± **SEM** | **Mean**± **SEM** | **Mean**± **SEM** | **P-value** | **FDR** |
| **Phylum** | | | | | |
| *Verrucomicrobiota* | 0.0±0.0 | 0.0±0.0 | 0.54±0.47 | 0.10 | 0.76 |
| *Fusobacteriota* | 3.51±1.69 | 3.15±0.9 | 4.24±1.33 | 0.39 | 0.83 |
| *Pseudomonadota* | 3.51±1.18 | 6.53±1.49 | 6.4±1.4 | 0.46 | 0.83 |
| *Bacteroidota* | 34.01±4.23 | 27.08±2.89 | 33.98±5.51 | 0.52 | 0.83 |
| *Bacillota* | 47.74±5.65 | 52.8±3.15 | 46.23±4.08 | 0.56 | 0.83 |
| *Thermodesulfobacteriota* | 0.39±0.2 | 0.23±0.06 | 0.19±0.12 | 0.62 | 0.83 |
| *Actinomycetota* | 10.49±5.65 | 8.69±2.29 | 6.79±1.93 | 0.76 | 0.87 |
| *Campylobacterota* | 0.31±0.12 | 1.47±0.66 | 1.58±1.09 | 0.93 | 0.93 |
| **Genus** | | | | | |
| *Phocaeicola* | 11.34±4.74 | 6.12±1.71 | 13.78±3.26 | 0.02* | 0.51 |
| *Mediterraneibacter* | 2.02±0.39 | 1.62±0.37 | 4.17±1.56 | 0.10 | 0.89 |
| *Holdemanella* | 2.81±1.23 | 1.26±0.88 | 0.18±0.15 | 0.12 | 0.89 |
| *Lachnoclostridium* | 1.17±0.25 | 1.17±0.19 | 1.69±0.28 | 0.21 | 0.89 |
| *Sutterella* | 1.1±0.45 | 1.46±0.48 | 4.53±1.75 | 0.31 | 0.89 |
| *Hoylesella* | 3.22±3.22 | 0.07±0.07 | 0.0±0.0 | 0.32 | 0.89 |
| *Megasphaera* | 2.25±0.89 | 0.97±0.35 | 0.77±0.39 | 0.33 | 0.89 |
| *Bifidobacterium* | 6.28±5.62 | 3.02±1.53 | 0.36±0.14 | 0.34 | 0.89 |
| *Fusobacterium* | 3.51±1.69 | 3.15±0.9 | 4.24±1.33 | 0.39 | 0.89 |
| *Bacteroides* | 5.15±2.18 | 6.24±1.87 | 6.14±1.48 | 0.41 | 0.89 |
| *Collinsella* | 3.54±1.18 | 4.25±1.14 | 5.5±2.06 | 0.44 | 0.89 |
| *Clostridium* | 1.94±1.28 | 1.45±0.45 | 0.52±0.28 | 0.54 | 0.92 |
| *Megamonas* | 4.16±1.55 | 3.47±1.3 | 4.22±1.52 | 0.60 | 0.92 |
| *Catenibacterium* | 4.03±2.58 | 2.56±1.03 | 0.51±0.34 | 0.65 | 0.92 |
| *Porphyromonas* | 1.03±0.82 | 3.31±1.64 | 3.16±3.16 | 0.69 | 0.92 |
| *Segatella* | 11.61±5.19 | 9.27±2.4 | 7.91±5.72 | 0.69 | 0.92 |
| *Peptoniphilus* | 1.47±1.09 | 3.38±1.05 | 1.86±1.52 | 0.71 | 0.92 |
| *Finegoldia* | 2.55±1.84 | 2.11±0.9 | 2.1±2.03 | 0.76 | 0.93 |
| *Blautia* | 9.09±2.18 | 9.63±2.01 | 7.66±1.57 | 0.81 | 0.94 |
| *Escherichia* | 0.7±0.61 | 4.66±1.57 | 1.68±0.97 | 0.91 | 0.96 |
| *Anaerococcus* | 2.22±1.74 | 4.0±1.58 | 7.04±4.62 | 0.92 | 0.96 |
| *Peptacetobacter* | 3.76±1.85 | 5.88±1.7 | 3.76±1.25 | 0.97 | 0.97 |
| **Species** | | | | | |
| *Bacteroides stercoris* | 3.75±1.82 | 1.2±0.32 | 4.07±1.45 | 0.06 | 0.67 |
| *Hoylesella timonensis* | 3.22±3.22 | 0.0±0.0 | 0.0±0.0 | 0.06 | 0.67 |
| *Phocaeicola vulgatus* | 2.74±0.99 | 2.73±1.59 | 3.74±1.53 | 0.12 | 0.67 |
| *Holdemanella porci* | 2.81±1.23 | 1.18±0.88 | 0.15±0.12 | 0.12 | 0.67 |
| *Ruminococcus torques* | 0.22±0.09 | 0.15±0.04 | 2.79±1.51 | 0.15 | 0.67 |
| *Phocaeicola coprocola* | 1.12±0.9 | 1.05±0.36 | 2.83±0.92 | 0.18 | 0.67 |
| *Lachnoclostridium*  *edouardi* | 0.85±0.17 | 0.85±0.15 | 1.35±0.29 | 0.21 | 0.67 |
| *Fusobacterium perfoetens* | 2.26±0.85 | 2.64±0.84 | 3.77±1.09 | 0.25 | 0.67 |
| *Phocaeicola massiliensis* | 1.68±0.96 | 0.75±0.31 | 5.93±3.06 | 0.27 | 0.67 |
| *Blautia schinkii* | 3.07±1.25 | 2.02±0.79 | 1.17±0.35 | 0.28 | 0.67 |
| *Collinsella intestinalis* | 2.79±1.15 | 3.07±1.05 | 3.45±1.12 | 0.31 | 0.67 |
| *Ruminococcus gnavus* | 1.36±0.38 | 1.22±0.36 | 0.88±0.29 | 0.31 | 0.67 |
| *Bifidobacterium pullorum* | 6.08±5.66 | 2.41±1.49 | 0.36±0.14 | 0.32 | 0.67 |
| *Megasphaera elsdenii* | 2.25±0.89 | 0.95±0.35 | 0.77±0.39 | 0.33 | 0.67 |
| *Phocaeicola plebeius* | 5.79±3.7 | 1.34±0.42 | 1.19±0.43 | 0.36 | 0.67 |
| *Sutterella massiliensis* | 1.1±0.45 | 1.15±0.43 | 4.25±1.81 | 0.37 | 0.67 |
| *Peptoniphilus olsenii* | 0.09±0.09 | 2.73±0.91 | 1.48±1.18 | 0.52 | 0.88 |
| *Megamonas funiformis* | 4.16±1.55 | 3.34±1.22 | 4.11±1.5 | 0.60 | 0.92 |
| *Catenibacterium*  *mitsuokai* | 4.03±2.58 | 2.56±1.03 | 0.51±0.34 | 0.65 | 0.92 |
| *Segatella copri* | 11.61±5.19 | 9.27±2.4 | 7.91±5.72 | 0.69 | 0.92 |
| *Blautia argi* | 1.3±0.37 | 1.32±0.23 | 1.6±0.45 | 0.72 | 0.92 |
| *Blautia hominis* | 2.1±0.74 | 2.52±0.86 | 1.87±0.86 | 0.75 | 0.92 |
| *Blautia caecimuris* | 2.0±0.6 | 2.68±0.72 | 1.21±0.26 | 0.76 | 0.92 |
| *Finegoldia magna* | 2.55±1.84 | 2.11±0.9 | 2.1±2.03 | 0.76 | 0.92 |
| *Bacteroides fragilis* | 0.11±0.07 | 3.16±1.68 | 0.65±0.59 | 0.83 | 0.97 |
| *Anaerococcus octavius* | 0.73±0.47 | 2.44±1.44 | 6.94±4.55 | 0.90 | 0.98 |
| *Escherichia fergusonii* | 0.69±0.59 | 4.62±1.56 | 1.68±0.97 | 0.91 | 0.98 |
| *Peptacetobacter*  *hiranonis* | 3.76±1.85 | 5.88±1.7 | 3.76±1.25 | 0.97 | 0.99 |
| *Porphyromonas somerae* | 0.38±0.38 | 1.77±1.33 | 2.84±2.84 | 0.99 | 0.99 |

Supplementary Figure S2: Alpha and beta diversity according to BCS. (a) Microbial richness and evenness were measured based on Shannon index. PCoA was performed based on un-weighted and weighted UniFrac distances. (b) List of alpha diversity indices. (c)

1. **(b)**


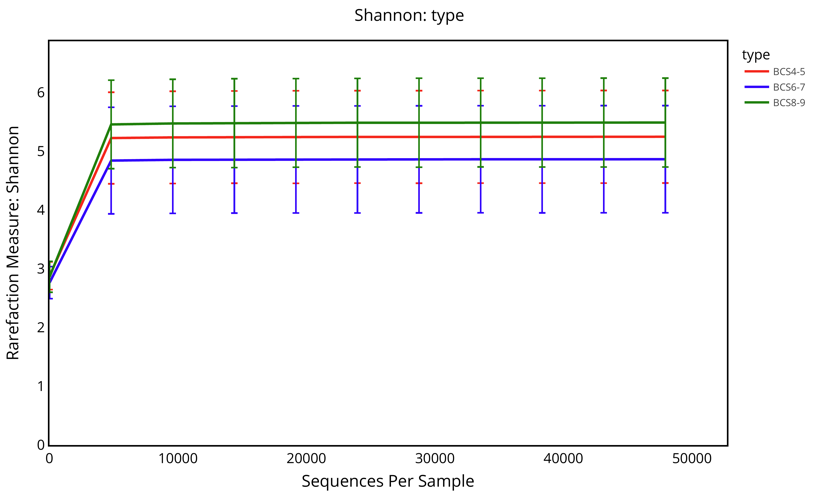

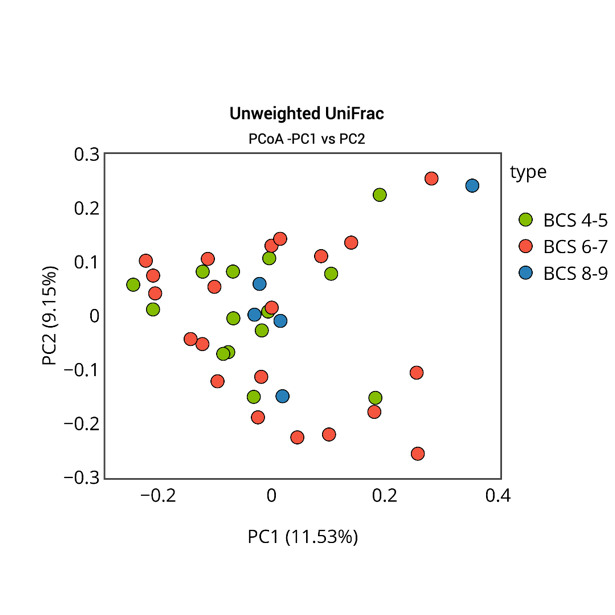


**(C)**

| **Diversity Index** | **BCS 4-5** | | **BCS 6-7** | | **BCS 8-9** | | **P-value** | **FDR** |
| --- | --- | --- | --- | --- | --- | --- | --- | --- |
|  | **Mean** | **SEM** | **Mean** | **SEM** | **MEAN** | **SEM** |  |  |
| ASVs | 196.79 | 17.78 | 180.24 | 14.60 | 253.40 | 19.31 | 0.05 | 0.22 |
| Shannon | 5.25 | 0.21 | 4.86 | 0.20 | 5.49 | 0.34 | 0.25 | 0.33 |
| Gini-Simpson | 0.94 | 0.01 | 0.92 | 0.01 | 0.93 | 0.02 | 0.36 | 0.36 |
| PD whole tree | 18.81 | 1.28 | 18.13 | 1.15 | 22.74 | 0.62 | 0.14 | 0.27 |

Supplementary Table S3. Relative abundance at the phylum, genus, and species levelaccording to BCS. Kruskal–Wallis test was used, and data are shown as the mean ± SEM. Major or significantly different bacterial groups (abundance ≥ 1% or P < 0.05) are shown. Asterisks indicate P < 0.05.

|  | **BCS 4-5** | **BCS 6-7** | **BCS8-9** |  | |
| --- | --- | --- | --- | --- | --- |
|  | **Mean**± **SEM** | **Mean**± **SEM** | **Mean**± **SEM** | **P-value** | **FDR** |
| **Phylum** | | | | | |
| *Verrucomicrobiota* | 0.0±0.0 | 0.0±0.0 | 0.86±0.74 | 0.01* | 0.08 |
| *Fusobacteriota* | 2.69±0.91 | 3.36±0.98 | 5.73±2.62 | 0.34 | 0.78 |
| *Bacteroidota* | 32.44±3.74 | 26.8±3.4 | 32.56±4.34 | 0.36 | 0.78 |
| *Thermodesulfobacteriota* | 0.28±0.09 | 0.27±0.08 | 0.06±0.04 | 0.39 | 0.78 |
| *Pseudomonadota* | 5.75±1.39 | 6.02±1.67 | 7.01±2.3 | 0.58 | 0.86 |
| *Bacillota* | 50.09±3.68 | 52.16±3.49 | 46.48±6.78 | 0.64 | 0.86 |
| *Actinomycetota* | 7.41±1.86 | 9.98±2.97 | 5.97±3.16 | 0.88 | 0.95 |
| *Campylobacterota* | 1.3±0.76 | 1.34±0.75 | 1.29±0.87 | 0.95 | 0.95 |
| **Genus** | | | | | |
| *Phocaeicola* | 11.69±3.3 | 5.7±1.66 | 10.79±2.42 | 0.06 | 0.82 |
| *Sutterella* | 1.57±0.79 | 1.26±0.32 | 6.46±2.51 | 0.14 | 0.82 |
| *Megamonas* | 3.6±1.31 | 4.6±1.48 | 0.39±0.33 | 0.20 | 0.82 |
| *Bifidobacterium* | 1.08±0.4 | 4.91±2.39 | 0.12±0.09 | 0.21 | 0.82 |
| *Lachnoclostridium* | 1.54±0.28 | 1.06±0.19 | 1.45±0.28 | 0.25 | 0.82 |
| *Finegoldia* | 0.61±0.52 | 3.42±1.3 | 1.32±1.21 | 0.32 | 0.82 |
| *Fusobacterium* | 2.69±0.91 | 3.36±0.98 | 5.73±2.62 | 0.34 | 0.82 |
| *Blautia* | 10.3±1.83 | 9.24±2.29 | 5.59±1.51 | 0.35 | 0.82 |
| *Clostridium* | 1.48±0.56 | 1.31±0.55 | 1.07±0.56 | 0.52 | 0.90 |
| *Mediterraneibacter* | 1.89±0.33 | 1.79±0.44 | 4.69±2.54 | 0.54 | 0.90 |
| *Bacteroides* | 4.4±1.11 | 7.26±2.3 | 5.63±1.33 | 0.55 | 0.90 |
| *Peptacetobacter* | 6.17±2.16 | 5.16±1.7 | 2.18±0.59 | 0.59 | 0.90 |
| *Anaerococcus* | 2.16±1.27 | 3.89±1.64 | 12.35±7.67 | 0.62 | 0.90 |
| *Catenibacterium* | 1.26±0.52 | 3.61±1.39 | 0.26±0.17 | 0.68 | 0.90 |
| *Peptoniphilus* | 2.39±1.37 | 3.01±1.04 | 2.97±2.37 | 0.73 | 0.90 |
| *Segatella* | 9.73±3.56 | 9.95±2.98 | 5.74±3.98 | 0.75 | 0.90 |
| *Collinsella* | 4.51±1.67 | 4.22±1.0 | 4.81±3.26 | 0.93 | 0.95 |
| *Escherichia* | 3.09±1.45 | 4.46±1.77 | 0.33±0.28 | 0.95 | 0.95 |
| *Porphyromonas* | 4.87±2.76 | 1.15±0.88 | 5.06±5.06 | 0.95 | 0.95 |
| **Species** | | | | | |
| *Ruminococcus torques* | 0.18±0.05 | 0.29±0.17 | 3.78±2.3 | 0.02* | 0.59 |
| *Bacteroides stercoris* | 2.78±0.99 | 1.33±0.45 | 3.9±1.57 | 0.12 | 0.86 |
| *Megamonas funiformis* | 3.6±1.31 | 4.4±1.37 | 0.39±0.33 | 0.20 | 0.86 |
| *Blautia argi* | 1.69±0.29 | 1.28±0.28 | 0.85±0.29 | 0.24 | 0.86 |
| *Bifidobacterium pullorum* | 0.66±0.29 | 4.39±2.36 | 0.11±0.09 | 0.24 | 0.86 |
| *Sutterella massiliensis* | 1.5±0.78 | 1.09±0.31 | 5.31±2.73 | 0.25 | 0.86 |
| *Fusobacterium perfoetens* | 2.16±0.66 | 2.56±0.84 | 5.68±2.65 | 0.25 | 0.86 |
| *Ruminococcus gnavus* | 1.28±0.3 | 1.26±0.42 | 0.48±0.14 | 0.30 | 0.86 |
| *Finegoldia magna* | 0.61±0.52 | 3.42±1.3 | 1.32±1.21 | 0.32 | 0.86 |
| *Blautia schinkii* | 2.37±0.67 | 2.09±0.95 | 0.69±0.21 | 0.37 | 0.86 |
| *Blautia hominis* | 2.27±0.53 | 2.72±1.07 | 0.81±0.3 | 0.39 | 0.86 |
| *Blautia caecimuris* | 2.66±0.86 | 2.4±0.72 | 0.74±0.28 | 0.43 | 0.86 |
| *Phocaeicola plebeius* | 3.18±1.66 | 1.3±0.49 | 1.47±0.41 | 0.46 | 0.86 |
| *Phocaeicola vulgatus* | 5.21±2.97 | 1.44±0.33 | 2.84±1.4 | 0.49 | 0.86 |
| *Phocaeicola coprocola* | 1.93±0.79 | 0.95±0.3 | 1.96±0.8 | 0.54 | 0.86 |
| *Phocaeicola massiliensis* | 1.07±0.46 | 1.96±1.18 | 4.16±2.48 | 0.59 | 0.86 |
| *Bacteroides fragilis* | 0.44±0.25 | 3.88±2.06 | 0.08±0.08 | 0.59 | 0.86 |
| *Peptacetobacter hiranonis* | 6.17±2.16 | 5.16±1.7 | 2.18±0.59 | 0.59 | 0.86 |
| *Peptoniphilus olsenii* | 1.69±1.11 | 2.15±0.86 | 2.93±2.37 | 0.67 | 0.88 |
| *Catenibacterium mitsuokai* | 1.26±0.52 | 3.61±1.39 | 0.26±0.17 | 0.68 | 0.88 |
| *Collinsella tanakaei* | 0.6±0.2 | 0.55±0.15 | 2.29±1.75 | 0.73 | 0.89 |
| *Segatella copri* | 9.73±3.56 | 9.95±2.98 | 5.74±3.98 | 0.75 | 0.89 |
| *Anaerococcus octavius* | 1.52±1.17 | 1.94±1.37 | 12.23±7.59 | 0.78 | 0.89 |
| *Porphyromonas somerae* | 3.28±2.43 | 0.11±0.11 | 4.55±4.55 | 0.90 | 0.95 |
| *Collinsella intestinalis* | 3.41±1.51 | 3.14±0.94 | 2.12±1.31 | 0.92 | 0.95 |
| *Escherichia fergusonii* | 3.03±1.42 | 4.46±1.77 | 0.33±0.28 | 0.95 | 0.95 |

Supplementary Figure S3: Alpha and beta diversity according to sex (a) Microbial richness and evenness were measured based on Shannon index. PCoA was performed based on un-weighted and weighted UniFrac distances. (b) List of alpha diversity indices. (c)

1. **(b)**


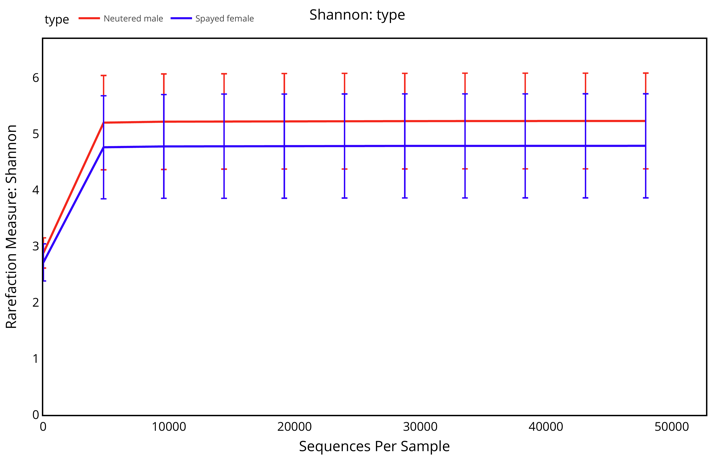

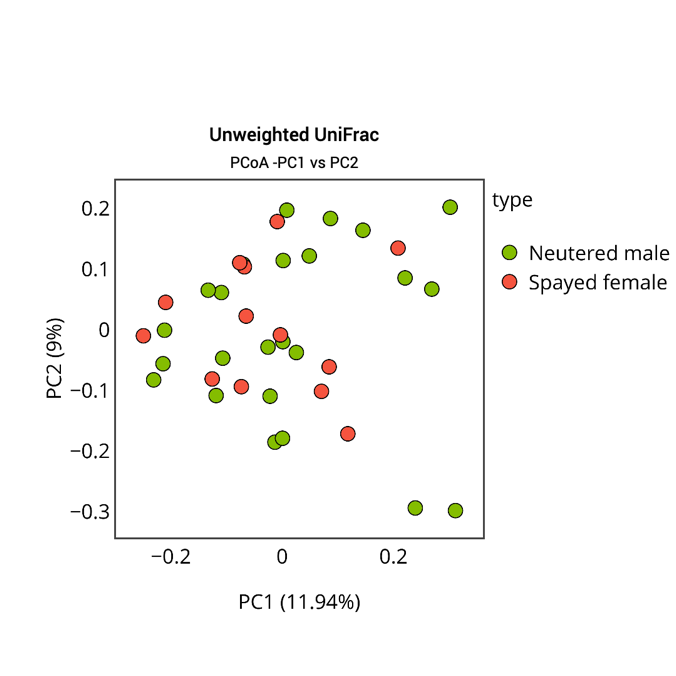


**(C)**

| **Diversity Index** | **Spayed female** | | **Male neutered** | | **95% Confidence Interval** | | **P-value** | **FDR** |
| --- | --- | --- | --- | --- | --- | --- | --- | --- |
|  | **Mean** | **SEM** | **Mean** | **SEM** | **Lower** | **Upper** |  |  |
| ASVs | 172.08 | 18.93 | 211.75 | 13.52 | -95.00 | 10.00 | 0.09 | 0.18 |
| Shannon | 4.79 | 0.26 | 5.23 | 0.17 | -1.06 | 0.19 | 0.17 | 0.20 |
| Gini-Simpson | 0.91 | 0.02 | 0.93 | 0.01 | -0.06 | 0.01 | 0.20 | 0.20 |
| PD whole tree | 16.94 | 1.48 | 20.37 | 0.92 | -7.53 | 0.38 | 0.08 | 0.18 |

Supplementary Table S4. Relative abundance at the phylum, genus, and species level according to sex. Wilcoxon Rank Sum test was used, and data are shown as the mean ± SEM. Major or significantly different bacterial groups (abundance ≥ 1% or P < 0.05) are shown. Asterisks indicate P < 0.05.

|  | **Spayed female** | **Male neutered** |  | |
| --- | --- | --- | --- | --- |
|  | **Mean± SEM** | **Mean± SEM** | **P-value** | **FDR** |
| **Phylum** | | | | |
| *Pseudomonadota* | 4.99±2.14 | 7.2±1.2 | 0.05* | 0.40 |
| *Verrucomicrobiota* | 0.0±0.0 | 0.18±0.16 | 0.20 | 0.69 |
| *Bacteroidota* | 26.64±4.75 | 32.29±2.39 | 0.39 | 0.69 |
| *Campylobacterota* | 1.06±0.68 | 1.57±0.71 | 0.39 | 0.69 |
| *Bacillota* | 51.26±4.12 | 48.46±2.85 | 0.50 | 0.69 |
| *Fusobacteriota* | 3.17±1.08 | 3.83±0.97 | 0.56 | 0.69 |
| *Thermodesulfobacteriota* | 0.32±0.11 | 0.24±0.06 | 0.68 | 0.69 |
| *Actinomycetota* | 12.5±4.78 | 6.19±1.07 | 0.69 | 0.69 |
| **Genus** | | | | |
| *Finegoldia* | 0.0±0.0 | 3.62±1.15 | 0.004* | 0.10 |
| *Sutterella* | 0.96±0.43 | 2.75±0.77 | 0.04 | 0.51 |
| *Peptoniphilus* | 1.45±0.87 | 3.87±1.14 | 0.07 | 0.54 |
| *Streptococcus* | 1.6±1.34 | 0.41±0.4 | 0.13 | 0.60 |
| *Bacteroides* | 4.26±1.42 | 7.73±1.92 | 0.16 | 0.60 |
| *Blautia* | 12.11±3.18 | 6.94±1.28 | 0.17 | 0.60 |
| *Escherichia* | 3.27±2.22 | 4.01±1.32 | 0.18 | 0.60 |
| *Collinsella* | 6.03±2.02 | 3.41±0.88 | 0.20 | 0.60 |
| *Anaerococcus* | 4.06±2.35 | 5.04±1.93 | 0.34 | 0.74 |
| *Catenibacterium* | 1.85±1.3 | 1.81±0.63 | 0.37 | 0.74 |
| *Turicibacter* | 2.08±2.08 | 0.0±0.0 | 0.39 | 0.74 |
| *Bifidobacterium* | 5.99±3.73 | 1.05±0.53 | 0.41 | 0.74 |
| *Helicobacter* | 0.69±0.62 | 1.41±0.71 | 0.43 | 0.74 |
| *Porphyromonas* | 3.02±2.14 | 3.27±1.71 | 0.45 | 0.74 |
| *Peptacetobacter* | 6.54±2.36 | 3.41±1.01 | 0.48 | 0.74 |
| *Megasphaera* | 1.02±0.5 | 1.2±0.38 | 0.52 | 0.74 |
| *Mediterraneibacter* | 1.8±0.3 | 2.16±0.6 | 0.52 | 0.74 |
| *Fusobacterium* | 3.17±1.08 | 3.83±0.97 | 0.56 | 0.74 |
| *Enterococcus* | 2.32±2.15 | 0.08±0.04 | 0.61 | 0.78 |
| *Lachnoclostridium* | 1.17±0.22 | 1.38±0.21 | 0.69 | 0.80 |
| *Clostridium* | 1.39±0.62 | 1.42±0.48 | 0.71 | 0.80 |
| *Megamonas* | 4.49±1.39 | 3.51±1.33 | 0.74 | 0.80 |
| *Phocaeicola* | 7.62±2.41 | 9.25±2.16 | 0.81 | 0.85 |
| *Segatella* | 10.2±4.42 | 8.45±2.31 | 1.00 | 1.00 |
| **Species** | | | | |
| *Finegoldia magna* | 0.0±0.0 | 3.62±1.15 | 0.004* | 0.11 |
| *Sutterella massiliensis* | 0.78±0.39 | 2.41±0.76 | 0.04* | 0.39 |
| *Ruminococcus gnavus* | 1.36±0.3 | 0.74±0.15 | 0.07 | 0.39 |
| *Anaerococcus*  *provencensis* | 0.76±0.76 | 1.25±0.69 | 0.08 | 0.39 |
| *Blautia schinkii* | 3.7±1.56 | 1.02±0.21 | 0.10 | 0.39 |
| *Bacteroides fragilis* | 1.07±0.85 | 3.08±1.79 | 0.12 | 0.39 |
| *Blautia caecimuris* | 2.65±0.75 | 1.73±0.56 | 0.12 | 0.39 |
| *Collinsella intestinalis* | 5.08±1.9 | 2.06±0.57 | 0.12 | 0.39 |
| *Bifidobacterium pullorum* | 5.98±3.73 | 0.4±0.15 | 0.15 | 0.40 |
| *Blautia hominis* | 3.67±1.64 | 1.41±0.35 | 0.18 | 0.40 |
| *Escherichia fergusonii* | 3.24±2.21 | 3.98±1.31 | 0.18 | 0.40 |
| *Peptoniphilus olsenii* | 1.29±0.76 | 2.77±0.98 | 0.19 | 0.40 |
| *Enterococcus hirae* | 2.14±2.14 | 0.0±0.0 | 0.19 | 0.40 |
| *Blautia argi* | 1.54±0.29 | 1.2±0.25 | 0.21 | 0.40 |
| *Phocaeicola coprocola* | 0.67±0.26 | 1.76±0.49 | 0.23 | 0.41 |
| *Catenibacterium*  *mitsuokai* | 1.85±1.3 | 1.81±0.63 | 0.37 | 0.60 |
| *Anaerococcus octavius* | 3.3±2.31 | 3.35±1.81 | 0.39 | 0.60 |
| *Fusobacterium perfoetens* | 2.23±0.73 | 3.36±0.91 | 0.40 | 0.60 |
| *Peptacetobacter*  *hiranonis* | 6.54±2.36 | 3.41±1.01 | 0.48 | 0.69 |
| *Megasphaera elsdenii* | 1.02±0.5 | 1.18±0.38 | 0.52 | 0.70 |
| *Phocaeicola plebeius* | 3.07±1.78 | 1.38±0.43 | 0.59 | 0.75 |
| *Megamonas funiformis* | 4.43±1.39 | 3.36±1.23 | 0.76 | 0.93 |
| *Porphyromonas somerae* | 2.82±1.97 | 1.43±1.34 | 0.79 | 0.93 |
| *Bacteroides stercoris* | 2.34±0.9 | 2.29±0.62 | 0.89 | 0.98 |
| *Phocaeicola vulgatus* | 2.34±0.75 | 3.51±1.75 | 0.91 | 0.98 |
| *Phocaeicola massiliensis* | 1.33±0.53 | 2.41±1.14 | 0.97 | 1.00 |
| *Segatella copri* | 10.2±4.42 | 8.45±2.31 | 1.00 | 1.00 |

Supplementary Figure S4: Alpha and beta diversity according to cat’s diet. (a) Microbial richness and evenness were measured using the Shannon index. PCoA was performed based on unweighted and weighted UniFrac distances. (b) List of alpha diversity indices. (c) Significant differences at the species level between BCS 4-5 and 8-9 groups are shown in panel. (d)

1. **(b)**


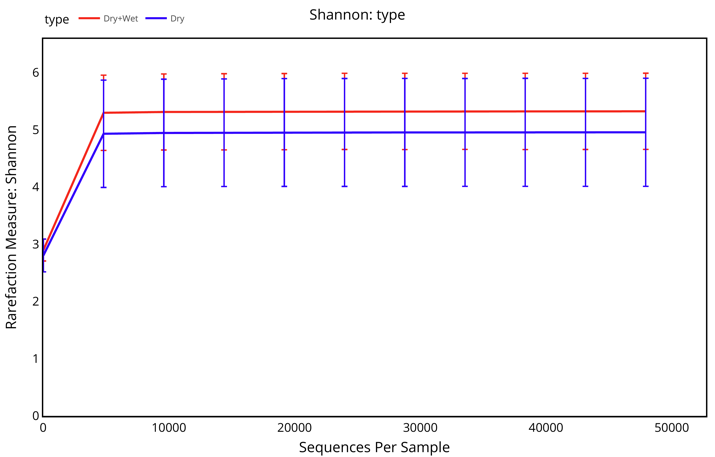

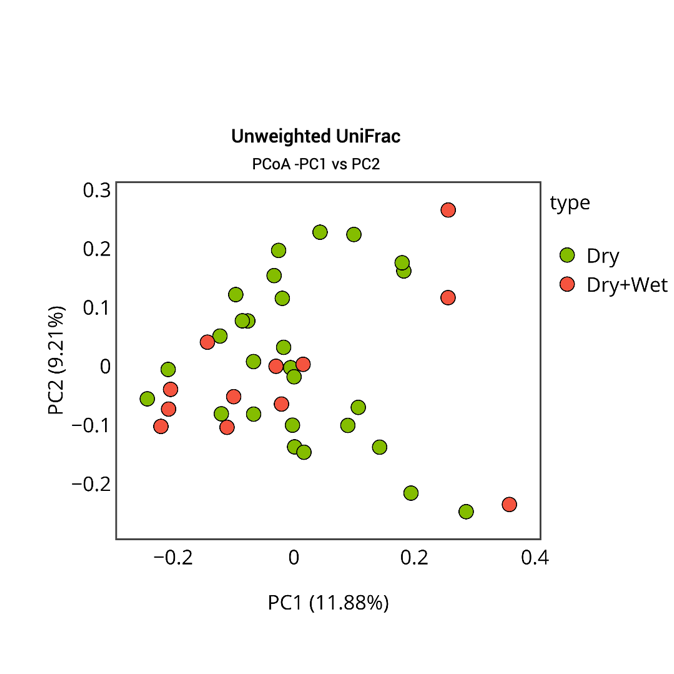


**(c)**

| **Diversity Index** | **Dry** | | **Dry+Wet** | | **95% Confidence Interval** | | **P-value** | **FDR** |
| --- | --- | --- | --- | --- | --- | --- | --- | --- |
|  | **Mean** | **SEM** | **Mean** | **SEM** | **Lower** | **Upper** |  |  |
| ASVs | 196.52 | 13.74 | 190.50 | 18.03 | -40.00 | 60.00 | 0.46 | 0.46 |
| Shannon | 4.96 | 0.18 | 5.32 | 0.19 | -0.96 | 0.32 | 0.31 | 0.46 |
| Gini-Simpson | 0.92 | 0.01 | 0.94 | 0.01 | -0.05 | 0.01 | 0.27 | 0.46 |
| PD whole tree | 19.29 | 1.00 | 17.92 | 1.31 | -2.33 | 5.54 | 0.37 | 0.46 |

**(d)**


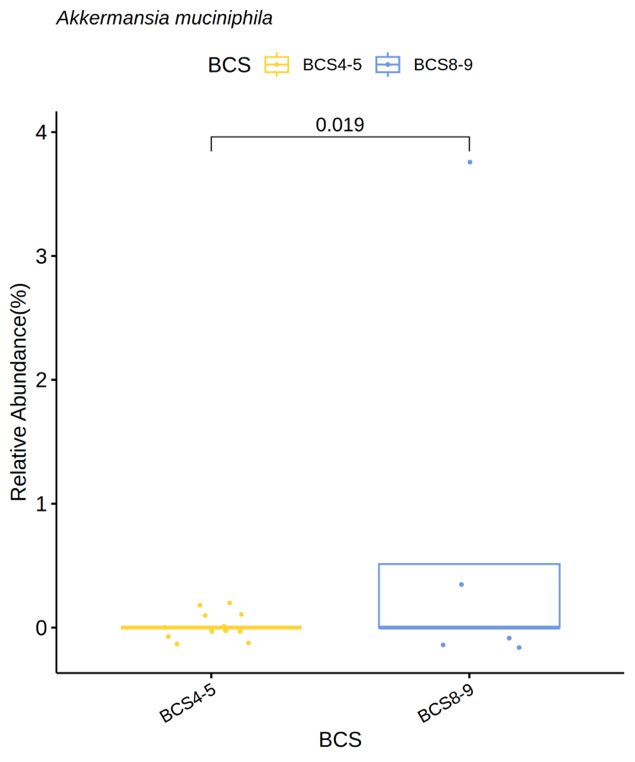


Supplementary Table S5. Relative abundance at the phylum, genus, and species level according to diet. Wilcoxon Rank Sum test was used, and data are shown as the mean ± SEM. Major or significantly different bacterial groups (abundance ≥ 1% or P < 0.05) are shown. Asterisks indicate P < 0.05.

|  | **Dry** | **Dry+Wet** |  | |
| --- | --- | --- | --- | --- |
|  | **Mean± SEM** | **Mean± SEM** | **P-value** | **FDR** |
| **Phylum** | | | | |
| *Campylobacterota* | 1.87±0.69 | 0.17±0.08 | 0.14 | 0.66 |
| *Bacteroidota* | 27.61±2.81 | 33.07±0.08 | 0.17 | 0.66 |
| *Verrucomicrobiota* | 0.16±0.14 | 0.00±0.00 | 0.25 | 0.66 |
| *Bacillota* | 49.87±2.89 | 52.91±4.53 | 0.50 | 0.89 |
| *Thermodesulfobacteriota* | 0.20±0.05 | 0.29±0.11 | 0.70 | 0.89 |
| *Fusobacteriota* | 3.78±0.91 | 2.49±0.95 | 0.70 | 0.89 |
| *Actinomycetota* | 10.11±2.48 | 5.40±0.99 | 0.78 | 0.89 |
| *Pseudomonadota* | 6.35±1.35 | 5.61±1.61 | 1.00 | 1.00 |
| **Genus** | | | | |
| *Blautia* | 6.92±1.46 | 14.47±2.68 | 0.003* | 0.07 |
| *Lachnoclostridium* | 1.04±0.15 | 1.79±0.29 | 0.04* | 0.41 |
| *Peptacetobacter* | 3.21±0.72 | 9.91±3.2 | 0.07 | 0.46 |
| *Clostridium* | 0.86±0.2 | 2.52±1.02 | 0.10 | 0.51 |
| *Anaerococcus* | 5.72±1.95 | 0.71±0.7 | 0.16 | 0.51 |
| *Mediterraneibacter* | 2.26±0.6 | 2.04±0.32 | 0.17 | 0.51 |
| *Phocaeicola* | 6.93±1.5 | 12.18±3.66 | 0.17 | 0.51 |
| *Segatella* | 7.97±2.29 | 13.23±4.29 | 0.21 | 0.55 |
| *Bacteroides* | 7.18±1.8 | 3.09±0.93 | 0.25 | 0.56 |
| *Peptoniphilus* | 3.27±0.94 | 1.39±1.38 | 0.27 | 0.56 |
| *Finegoldia* | 2.53±1.0 | 0.62±0.6 | 0.35 | 0.66 |
| *Escherichia* | 3.95±1.39 | 2.63±1.7 | 0.42 | 0.70 |
| *Catenibacterium* | 2.52±1.07 | 2.23±0.95 | 0.44 | 0.70 |
| *Bifidobacterium* | 3.89±1.88 | 1.15±0.45 | 0.51 | 0.77 |
| *Collinsella* | 4.72±1.21 | 3.81±0.85 | 0.58 | 0.81 |
| *Megamonas* | 3.93±1.22 | 3.54±1.36 | 0.70 | 0.81 |
| *Fusobacterium* | 3.78±0.91 | 2.49±0.95 | 0.70 | 0.81 |
| *Megasphaera* | 1.28±0.38 | 0.87±0.33 | 0.73 | 0.81 |
| *Sutterella* | 2.06±0.64 | 1.87±0.9 | 0.74 | 0.81 |
| *Porphyromonas* | 3.13±1.42 | 2.68±2.67 | 0.89 | 0.91 |
| *Holdemanella* | 1.36±0.86 | 1.19±0.65 | 0.91 | 0.91 |
| **Species** | | | | |
| *Blautia schinkii* | 0.94±0.19 | 4.41±1.62 | 0.004* | 0.10 |
| *Blautia hominis* | 2.0±0.82 | 3.19±0.68 | 0.02* | 0.15 |
| *Lachnoclostridium*  *edouardi* | 0.76±0.14 | 1.37±0.21 | 0.02* | 0.15 |
| *Clostridium perfringens* | 0.26±0.1 | 1.84±1.02 | 0.02* | 0.15 |
| *Peptacetobacter*  *hiranonis* | 3.21±0.72 | 9.9±3.2 | 0.07 | 0.33 |
| *Blautia caecimuris* | 1.69±0.5 | 3.64±1.11 | 0.07 | 0.33 |
| *Ruminococcus gnavus* | 1.09±0.35 | 1.35±0.29 | 0.12 | 0.47 |
| *Peptoniphilus olsenii* | 2.57±0.8 | 1.17±1.17 | 0.15 | 0.50 |
| *Blautia argi* | 1.3±0.25 | 1.59±0.21 | 0.16 | 0.50 |
| *Anaerococcus octavius* | 4.47±1.89 | 0.0±0.0 | 0.20 | 0.54 |
| *Segatella copri* | 7.97±2.29 | 13.23±4.29 | 0.21 | 0.54 |
| *Phocaeicola vulgatus* | 1.81±0.53 | 5.37±3.38 | 0.30 | 0.70 |
| *Finegoldia magna* | 2.53±1.0 | 0.62±0.6 | 0.35 | 0.72 |
| *Phocaeicola plebeius* | 1.28±0.39 | 3.72±1.9 | 0.38 | 0.72 |
| *Fusobacterium*  *perfoetens* | 3.27±0.83 | 1.6±0.6 | 0.38 | 0.72 |
| *Escherichia fergusonii* | 3.93±1.39 | 2.6±1.68 | 0.42 | 0.72 |
| *Catenibacterium*  *mitsuokai* | 2.52±1.07 | 2.23±0.95 | 0.44 | 0.72 |
| *Collinsella intestinalis* | 3.41±1.04 | 2.53±0.69 | 0.46 | 0.72 |
| *Bacteroides fragilis* | 3.21±1.62 | 0.1±0.07 | 0.51 | 0.75 |
| *Phocaeicola coprocola* | 1.38±0.36 | 1.64±0.78 | 0.68 | 0.92 |
| *Megamonas funiformis* | 3.77±1.14 | 3.54±1.36 | 0.70 | 0.92 |
| *Bifidobacterium*  *pullorum* | 3.53±1.86 | 0.55±0.32 | 0.73 | 0.92 |
| *Megasphaera elsdenii* | 1.27±0.38 | 0.84±0.34 | 0.77 | 0.93 |
| *Bacteroides stercoris* | 2.16±0.56 | 1.74±0.87 | 0.81 | 0.93 |
| *Phocaeicola massiliensis* | 2.28±1.01 | 1.29±0.57 | 0.86 | 0.93 |
| *Porphyromonas*  *somerae* | 1.44±0.97 | 2.67±2.67 | 0.87 | 0.93 |
| *Holdemanella porci* | 1.28±0.86 | 1.19±0.65 | 0.97 | 0.98 |
| *Sutterella massiliensis* | 1.68±0.61 | 1.86±0.9 | 0.98 | 0.98 |
